# The adaptation process of the Brazilian National Health System to the effects of climate change

**DOI:** 10.1590/S2237-96222026v35e20250665.en

**Published:** 2026-03-09

**Authors:** Klauss K. S. Garcia, Marco Aurélio Pereira Horta

**Affiliations:** ¹ London School of Hygiene & Tropical Medicine Faculty of Epidemiology and Population Health, Londres, Reino Unido.; ² Instituto Oswaldo Cruz, Rio de Janeiro, RJ, Brasil

## Introduction

The term “climate change” refers to alterations in long-term temperature and weather patterns, mostly driven by human activities such as the burning of fossil fuels. Climate change manifests through rising global temperatures, changes in precipitation cycles, and the increasing frequency of extreme weather events, such as floods, droughts, heat waves, land and rockslides, strong winds, storms, and wildfires ^1^. 

The health impacts of climate change are unevenly distributed and disproportionately affect countries and populations that have historically contributed less to greenhouse gas emissions ^2^. In Brazil, socially vulnerable groups-including Indigenous peoples, riverside communities, *quilombola* populations, and residents of peripheral urban areas-face a compounded burden arising both from climate risks and from systemic barriers to health care access. This dual exposure highlights an urgent scenario of climate injustice and a potential public health crisis ^3^.

Climate change exacerbates health conditions such as respiratory and allergic diseases associated with air pollution and increases cardiovascular morbidity and mortality due to heat stress and exposure to particulate matter. These changes also contribute to the decompensation of diabetes and to emergencies related to chronic kidney disease-both associated with dehydration-and increase occupational risks, particularly in the agricultural sector. Furthermore, worsening of mental health disorders has been reported as a result of extreme weather events and the insecurity caused by climate-related disasters. More frequent and intense heat waves have been recorded, leading to higher rates of heat stroke and cardiovascular complications ^4^.

Beyond individual health effects, climate change impacts broader environmental dimensions by intensifying droughts and floods. In 2024, Brazil experienced severe flooding in the South region ^5^ and intense droughts in the North ^6^. Specifically in Rio Grande do Sul, floods affected 96% of the state’s municipalities, impacting 2,400,000 people and resulting in more than 180 deaths, along with an increase in waterborne diseases such as leptospirosis ^5^. 

In the North, droughts have compromised access to water, food, and medicines, as well as transportation systems ^6^, which is particularly critical since many areas in the Amazon region depend on rivers as their main transportation routes. These conditions disproportionately affect Indigenous and riverside populations living in remote areas of the region ^6^. In the state of Amazonas alone, in 2024, droughts and dry periods affected more than 860,000 people ^7^. 

At the same time, large-scale wildfires also occurred across the country, particularly in the Central-West and Northern regions, including the Pantanal, the Cerrado, and the Amazon rainforest. Fires contribute to an increase in respiratory diseases by reducing air quality; moreover, they trigger migratory movements, as populations are forced to relocate in search of infrastructure and safe drinking water ^4^ ^,^ ^8^. Despite their different causes-whether accidental or intentional-these fires directly affected more than 18,900,000 people in Brazil in 2024 ^8^. 

All these events were so devastating that the Brazilian government established a national situation room for wildfire prevention and control, coordinated by the Office of the Chief of Staff and the Ministry of the Environment and Climate Change. Subsequently, the Ministry of Health implemented a national health emergency situation room on climate-related events, as a response measure to contain the consequences of the disaster. 

The 1988 Federal Constitution recognizes health as a fundamental right and establishes that it is the duty of the State to ensure conditions for its fulfillment ^9^. In view of the growing threats that climate change poses to this right, this article discusses key elements to strengthen political dialogue and guide the adaptation of the Brazilian National Health System (*Sistema Único de Saúde*, SUS).

### Developing climate resilience in SUS

Climate resilience is the capacity of ecological, social, or infrastructure systems to anticipate, absorb, adapt to, and recover from the impacts of climate change and extreme events ^10^. It involves prevention, diagnosis, and treatment actions, along with strengthening health education, particularly among managers. This process also expands the institutional autonomy of federative entities and adopts integrated and multisectoral strategies to reduce vulnerabilities and ensure the functioning of health systems, even during crises ^10^.

Within SUS, developing climate resilience requires strategic, structural, and intersectoral actions ^10^. The principle of equity must guide this process, prioritizing the needs of vulnerable and marginalized populations ^2^. State responsibility for providing health care does not exclude the role of individuals, families, companies, and society at large, highlighting the importance of social participation and political mobilization for systemic change ^9^.

In this context, “adaptation” in health systems can be understood as the process of adjusting structures, services, and practices based on lessons learned from previous events, thereby enhancing the capacity to respond to future climatic events-whether predictable or unexpected-and reducing their effects ^10^. In the current scenario, adapting SUS to the effects of climate change begins with strengthening its resilience. 

**Figure 1.**
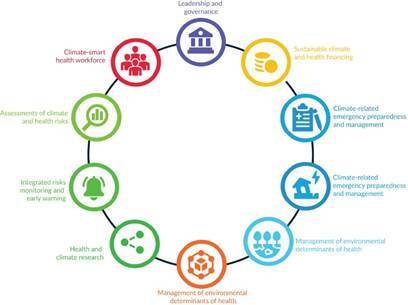
Components for developing climate-resilient and low-carbon health systems

Adapting and strengthening SUS in the face of climate change is aligned with global agendas and represents a commitment to human rights ^11^. The World Health Organization recommends that the climate resilience of health systems be built sustainably, focusing on components such as “Leadership and governance,” “Sustainable financing,” “Workforce and infrastructure,” “Research and technological development,” and “Health surveillance.” These components encompass actions related to health management, assessment, monitoring, preparedness, and emergency response (Figure 1). The use of these World Health Organization recommendations can serve as a guiding framework for organizing local health services during the adaptation process ^10^.

The adaptation process should begin with risk mapping that integrates climate and epidemiological data, identifying the regions and populations most exposed. The process includes developing early warning systems based on predictive risk analyses, with the potential use of artificial intelligence tools to enhance response speed. These systems should be implemented primarily in areas at high risk of floods, water scarcity, disease outbreaks, and heat waves. They should monitor indicators such as river levels, precipitation, temperature, and morbidity and mortality due to heat stress or climate-sensitive diseases (e.g., arboviruses). Monitoring these indicators will support public policies for rapid resource allocation and preparedness for climate-related emergencies ^12^.

The adaptation process must also prioritize health sector infrastructure. Hospitals and health centers need modifications to continue operating during floods, blackouts, droughts, and extreme heat. This includes alternative water sources, independent renewable energy, thermal insulation, strategic location outside high-risk areas, and safe access routes for health professionals ^13^. The loss of hospital functionality during floods in Rio Grande do Sul highlights the impact of insufficient local resilience during extreme events ^14^. Training of health professionals and teams is essential and fosters local empowerment. Rapid response protocols should be developed, particularly for climate-related emergencies, including strategies for population displacement and mental health support for affected communities ^15^.

Surveillance systems must be strengthened to monitor the spread of climate-sensitive diseases, including the enhancement of laboratory surveillance with biosafety level 2 and 3 facilities. Investment in scientific research is required to develop rapid diagnostic tests, enabling proactive responses to emerging and recurrent infectious diseases ^16^.

Water security also occupies a central role in the adaptation process. Droughts in the Amazon, which cause water scarcity and forced migrations, and floods in Rio Grande do Sul, which result in a lack of energy for water pumping, have revealed the direct connection between environmental degradation, climate change, and health outcomes ^17^. Investment is necessary in local water storage and treatment systems for isolated communities, the adoption of technologies such as desalination where feasible, and the protection of watersheds and native forests, which function as natural climate regulators. Logistical protocols for transporting water and patients must be coordinated among the health sector, the Armed Forces, and Civil Defense, especially for hard-to-reach communities, including Indigenous populations ^17^.

Urban planning must also integrate health into the adaptation process. In cities, rising temperatures and rainfall variability demand more green areas, infrastructure to mitigate urban heat islands, and expanded sanitation coverage. Migration from climate-impacted rural areas to urban zones requires coordinated social and urban policies to prevent the amplification of health risks in peripheral neighborhoods ^18^.

Public engagement and environmental and health education are pillars of effective adaptation. Community initiatives, accessible campaigns, and the inclusion of climate and health content in school curricula are key strategies. Local leadership, especially in Indigenous and riverside communities, should play a central role in formulating and implementing solutions adapted to their contexts and traditional knowledge ^19^.

### Barriers and pathways to adaptation

It is necessary to address the political, social, and economic challenges that must be overcome for the adaptation of SUS. In 2025, the environmental protection agenda faces pressure from economic groups linked to polluting and resource-intensive sectors such as agribusiness, deforestation, and mining, which impose barriers to prioritizing a strong climate agenda and decarbonization ^20^.

On the social front, historical inequality is manifested in the precariousness of services in peripheral territories and among Indigenous, riverside, and *quilombola* populations, who remain on the margins of formal health care networks and face logistical, cultural, and linguistic barriers to access ^21^. These conditions deepen vulnerability and limit the effectiveness of adaptation actions.

Furthermore, scientific denial represents a significant barrier to adaptation. As observed during the COVID-19 pandemic, critical or skeptical views regarding climate change reduce risk perception and the urgency of mitigation and adaptation measures ^22^. These narratives, often associated with populist discourse, circulate both among the general population and political leaders, potentially delaying or even paralyzing institutional advances in the health sector. To face this challenge, it is essential to strengthen health communication strategies that are transparent, participatory, and continuous, capable of combating misinformation, engaging society, and sustaining the legitimacy of SUS adaptation policies ^23^.

From an economic perspective, one of the main barriers is insufficient national funding, which is associated with rigid fiscal limits that hinder the creation of permanent investment mechanisms for resilient infrastructure and surveillance within SUS ^24^. Therefore, the creation of a national adaptation fund is urgent, with specific allocations for the health, urban development, and environmental protection sectors. 

Specifically for the health sector, a National Adaptation Plan should be developed and implemented to mobilize and distribute resources across federal, state, and municipal levels. These funds should encourage subnational authorities to design territorialized adaptation strategies that address the specific vulnerabilities and capacities of each locality. State and municipal health departments should assume leadership in decentralized implementation, advancing independently of the pace of the federal government.

### Pathways to adaptation

Clear adaptation targets must be integrated into medium- and long-term planning within the National Health Plan and should preferably align with the guidelines established in the Multi-Year Plan. Another key aspect is the incorporation of climate data into routine health surveillance and service preparedness protocols ^12^. Effective adaptation requires integration among health surveillance, primary care, specialized care, educational systems, academic institutions, and civil society, enabling a coordinated and intersectoral response that reflects Brazil’s territorial diversity ^10^.

Some actions that strengthen the adaptation process within SUS have already been initiated. In 2024, Brazil’s Ministry of Health established a working group for the development of the Health Sector Climate Change Adaptation Plan. In 2025, the organization continues to develop the Health Sector Adaptation Plan, focusing on health surveillance, health care, health promotion, and science, technology, innovation, and production in the sector. 

Adaptation experiences that affect health are not exclusive to the health sector and may require intersectoral negotiations and policies involving ministries such as the Ministry of the Environment and Climate Change and the Ministry of Cities, which further increases the complexity of the process. Interventions aimed at urban infrastructure, green and sustainable cities, and improvements in sanitation and environmental education have already been reported in the scientific literature as contributing to population health in contexts of climate-related impacts ^25^. 

It is essential to establish a robust, sustainable, and climate-informed health surveillance agenda, with continuous monitoring of epidemiological and environmental indicators such as temperature, precipitation, and river levels. Local epidemiological surveillance teams must be trained to interpret the relationships between climatic variables and health outcomes, as well as to anticipate extreme events such as floods and river droughts, in order to enable robust planning and timely response.

Building climate resilience within SUS is an urgent and complex public health challenge that Brazil faces-and one that will persist for years to come. This process requires the integration of meteorological, environmental, and epidemiological data; multiscale and long-term planning; and consistent, sustained strategic investments.

Although political support and federal leadership are essential for mobilizing resources and defining policy guidelines, the core of effective adaptation lies in empowering states, municipalities, and civil society as central actors. Their ability to provide rapid and appropriate responses to socially vulnerable populations is crucial.

Achieving climate resilience will require political commitment, territorial planning, and continuous intersectoral collaboration to ensure the fulfillment of SUS’s constitutional mandate and safeguard the right to health in a changing climate.
