# Supplementary material for: The adaptation process of the Brazilian National Health System to the effects of climate change
Source: Epidemiol Serv Saude. 2026 Mar 9;35:e20250665. doi: 10.1590/S2237-96222026v35e20250665.en (PMC12970837; doi:10.1590/S2237-96222026v35e20250665.en)
Supplement: Translation

# Translation: O processo de adaptação do Sistema Único de Saúde aos efeitos das mudanças climáticas

**DOI:** 10.1590/S2237-96222026v35e20250665.pt

**Authors:** Klauss K. S. Garcia, Marco Aurélio Pereira Horta

**Affiliations:** ¹London School of Hygiene & Tropical Medicine Faculty of Epidemiology and Population Health, Londres, Reino Unido.; ²Instituto Oswaldo Cruz, Rio de Janeiro, RJ, Brasil

Introdução

O termo “mudanças climáticas” se refere a alterações na temperatura e nos padrões climáticos de longo prazo, majoritariamente impulsionadas por atividades humanas, como a queima de combustíveis fósseis. As mudanças climáticas se manifestam através do aumento das temperaturas globais, de alterações nos ciclos de precipitação e na maior frequência com que eventos climáticos extremos ocorrem, a exemplo de enchentes, secas, ondas de calor, deslizamentos de terra/rochas, ventos intensos, tempestades e incêndios florestais ^1^. 

Os impactos das mudanças climáticas na saúde são distribuídos de forma desigual e afetam de modo desproporcional países e populações que historicamente contribuíram menos com emissões de gases de efeito estufa ^2^. No Brasil, grupos socialmente vulneráveis (incluindo povos indígenas, comunidades ribeirinhas, quilombolas e moradores de áreas urbanas periféricas) enfrentam um fardo agravado tanto pelos riscos climáticos quanto por barreiras sistêmicas ao acesso à saúde. Essa dupla exposição destaca um cenário urgente de injustiça climática e potencial crise sanitária ^3^.

As mudanças climáticas intensificam agravos à saúde, como doenças respiratórias e alérgicas associadas à poluição atmosférica e aumento da morbimortalidade cardiovascular por estresse térmico e exposição a materiais particulados. Tais mudanças também impactam descompensações do diabetes e emergências por doença renal crônica - ambas relacionadas à desidratação do organismo do indivíduo - e aumentam riscos ocupacionais, sobretudo no setor da agricultura. Além disso, relata-se agravamento de transtornos mentais por conta de eventos climáticos extremos e da insegurança gerada por desastres associados ao clima. Ondas de calor mais frequentes e intensas são registradas, o que eleva os casos de insolação e agravos cardiovasculares ^4^.

Além dos agravos em nível individual, as mudanças climáticas repercutem em dimensões ambientais mais amplas com a intensificação de secas e enchentes. Em 2024, o Brasil enfrentou enchentes severas na região Sul ^5^ e secas intensas na região Norte ^6^. Especificamente no Rio Grande do Sul, as enchentes atingiram 96% dos municípios do estado, com 2,4 milhões de pessoas afetadas e mais de 180 óbitos, além de ter aumentado a transmissão de doenças de veiculação hídrica como a leptospirose ^5^. 

No Norte, as secas comprometem o acesso a água, alimentos e medicamentos, além de comprometer os sistemas de transporte ^6^, o que é particularmente crítico, uma vez que diversas áreas da Amazônia dependem dos rios como principais vias de transporte. Essas condições afetam de forma desproporcional as populações indígenas e ribeirinhas residentes em áreas remotas da região ^6^. Somente no Amazonas, em 2024, as secas e estiagens afetaram mais de 860 mil pessoas ^7^. 

Simultaneamente, incêndios florestais também ocorreram em larga escala no país, principalmente nas regiões Centro-Oeste e Norte, ou seja, no Pantanal, no Cerrado e na floresta amazônica. As queimadas contribuem com o aumento de doenças respiratórias por diminuírem a qualidade do ar; além disso, desencadeiam movimentos migratórios, pois as populações se deslocam em busca de infraestrutura e água potável ^4^ ^,^ ^8^. Apesar das diferentes origens, sejam os incêndios acidentais, sejam incêndios intencionais, seus efeitos afetaram diretamente mais de 18,9 milhões de pessoas no país em 2024 ^8^. 

Todos esses eventos foram tão devastadores que o governo brasileiro instaurou uma sala de situação nacional para prevenção e controle de incêndios, presidida pela Casa Civil e pelo Ministério do Meio Ambiente e Mudança do Clima. Posteriormente, o Ministério da Saúde implementou uma sala de situação nacional de emergências climáticas em saúde, como medida de resposta para a contenção das consequências do evento. 

A Constituição Federal de 1988 considera que a saúde é um direito fundamental e destaca que é dever do Estado garantir condições para sua realização ^9^. Diante das ameaças crescentes das mudanças climáticas a esse direito, este artigo discutiu elementos centrais para fortalecer o diálogo político e orientar a adaptação do Sistema Único de Saúde (SUS).

Desenvolvendo resiliência climática no SUS

Resiliência climática é a capacidade de sistemas ecológicos, sociais ou de infraestrutura anteciparem, absorverem, adaptarem-se e recuperarem-se dos impactos das mudanças climáticas e de eventos extremos ^10^. Envolve ações de prevenção, diagnóstico e tratamento, além do fortalecimento da educação em saúde, especialmente entre gestores. O processo também amplia a autonomia institucional dos entes federativos e adota estratégias integradas e multissetoriais para reduzir vulnerabilidades e manter a operação dos sistemas de saúde, mesmo diante de crises ^10^.

No SUS, o desenvolvimento da resiliência climática exige ações estratégicas, estruturais e intersetoriais ^10^. O princípio da equidade deve orientar esse processo, priorizando as necessidades de populações vulneráveis e vulnerabilizadas ^2^. A responsabilidade estatal na oferta de cuidados de saúde não exclui o papel de indivíduos, famílias, empresas e da sociedade, destacando a importância da participação social e mobilização política para mudanças sistêmicas ^9^.

Nesse contexto, pode-se entender “adaptação” em sistemas de saúde como o processo de ajustar estruturas, serviços e práticas, com base em lições de eventos anteriores, amplificando a capacidade de resposta a eventos climáticos futuros - previsíveis ou inesperados - e diminuindo seus efeitos ^10^. No cenário atual, a adaptação do SUS aos efeitos das mudanças climáticas começa pelo fortalecimento de sua resiliência. 

Adaptar e fortalecer o SUS diante das mudanças climáticas está alinhado às agendas globais e representa um compromisso com os direitos humanos ^11^. A Organização Mundial da Saúde orienta que a resiliência climática de sistemas de saúde seja feita de forma sustentável e baseada em componentes que focam “Liderança e governança”, “Financiamento sustentável”, “Força de trabalho e infraestrutura”, “Pesquisa e desenvolvimento tecnológico” e “Vigilância em saúde”. Tais componentes englobam ações de gestão em saúde, avaliação, monitoramento, preparação e resposta a emergências (Figura 1). A utilização dessas recomendações da Organização Mundial da Saúde pode ser norteadora para a organização de serviços locais de saúde durante o processo de adaptação ^10^.

O processo de adaptação deve começar com o mapeamento de riscos que integre dados climáticos e epidemiológicos, identificando regiões e populações mais expostas. O processo inclui o desenvolvimento de sistemas de alerta precoce baseados em análises preditivas de risco, com o potencial uso de ferramentas de inteligência artificial para maior velocidade de resposta. Esses sistemas devem ser instalados prioritariamente em áreas com risco elevado de enchentes, escassez hídrica, surtos de doenças e ondas de calor, através do monitoramento de indicadores como níveis previstos de rios, precipitação, temperatura e morbimortalidade por estresse térmico ou doenças sensíveis ao clima (como arboviroses). O monitoramento desses indicadores subsidiará políticas públicas de alocação rápida de recursos e preparação para respostas a emergências relacionadas ao clima ^12^.

**Figura 1.** Componentes para o desenvolvimento de sistemas de saúde resilientes ao clima e de baixo carbono

O processo de adaptação também deve priorizar a infraestrutura do setor saúde. Hospitais e unidades básicas precisam de adaptações para seguir operando durante enchentes, apagões, secas e calor extremo. Isso inclui fontes alternativas de água, energia renovável independente, isolamento térmico, localização estratégica fora de áreas de risco e rotas seguras de acesso para profissionais ^13^. A perda de funcionalidade hospitalar nas enchentes do Rio Grande do Sul evidencia o impacto da falta de resiliência local no momento do evento ^14^. A capacitação dos profissionais e das equipes de saúde é fundamental e incentiva empoderamento local. Protocolos de resposta rápida devem ser produzidos, especialmente para emergências climáticas, incluindo estratégias para deslocamentos populacionais e apoio à saúde mental de comunidades afetadas ^15^.

Os sistemas de vigilância devem ser fortalecidos para monitorar a disseminação de doenças sensíveis ao clima, o que inclui o fortalecimento de vigilância laboratorial com unidades de biossegurança níveis 2 e 3. Deve-se investir em pesquisas científicas para o desenvolvimento de testes diagnósticos rápidos, com o objetivo de responder proativamente a doenças infecciosas novas e recorrentes ^16^.

A segurança hídrica também tem posição central no processo de adaptação. A seca na Amazônia, com escassez de água e migrações forçadas, e as enchentes no Rio Grande do Sul, com falta de energia para bombeamento de água, revelaram a conexão direta entre degradação ambiental, mudanças climáticas e desfechos em saúde ^17^. É necessário investir em sistemas locais de armazenamento e tratamento de água em comunidades isoladas, adotar tecnologias como a dessalinização, quando viável, e proteger bacias hidrográficas e florestas nativas, que funcionam como reguladores climáticos. Protocolos logísticos para transporte de água e pacientes devem ser articulados entre o setor saúde, as Forças Armadas e a Defesa Civil, especialmente para comunidades de difícil acesso, incluindo populações indígenas ^17^.

O planejamento urbano também deve integrar a saúde no processo de adaptação. Nas cidades, o aumento das temperaturas e a variabilidade da chuva demandam mais áreas verdes, infraestrutura para mitigar ilhas de calor e maior cobertura sanitária. A migração de áreas rurais impactadas pelo clima para zonas urbanas exige políticas sociais e urbanas coordenadas, evitando a ampliação dos riscos sanitários nas periferias ^18^.

O engajamento público e a educação ambiental e em saúde são pilares de uma adaptação eficaz. Iniciativas comunitárias, campanhas acessíveis e a inclusão de conteúdos sobre clima e saúde nos currículos escolares são estratégias-chave. Lideranças locais, sobretudo em comunidades indígenas e ribeirinhas, devem ter protagonismo na formulação e implementação de soluções adaptadas a seus contextos e saberes ^19^.

Barreiras e caminhos para a adaptação

Faz-se necessário comentar sobre os desafios e obstáculos políticos, sociais e econômicos que precisam ser superados para a adaptação do SUS. Em 2025, a agenda de proteção ambiental esbarra na pressão de grupos econômicos ligados a setores poluentes e de uso intensivo de recursos naturais como o agronegócio, o desmatamento e a mineração, impondo barreiras à priorização de uma agenda climática firme na descarbonização ^20^.

No campo social, a desigualdade histórica se manifesta na precariedade de serviços em territórios periféricos e populações indígenas, ribeirinhas e quilombolas, que permanecem à margem das redes formais de atenção e enfrentam barreiras logísticas, culturais e linguísticas no acesso à saúde ^21^. Essas condições aprofundam a vulnerabilidade e limitam a efetividade das ações de adaptação.

Além disso, o negacionismo científico constitui barreira relevante à adaptação. Tal como observado durante a pandemia de covid-19, visões críticas ou descrentes em relação às mudanças climáticas reduzem a percepção de risco e a urgência de medidas de mitigação e adaptação ^22^. Essas narrativas, frequentemente associadas a discursos populistas, circulam tanto na população em geral quanto entre lideranças políticas, podendo retardar ou até paralisar avanços institucionais no setor saúde. Para enfrentar esse desafio, é essencial fortalecer estratégias de comunicação em saúde que sejam transparentes, participativas e contínuas, capazes de combater a desinformação, engajar a sociedade e sustentar a legitimidade das políticas de adaptação do SUS ^23^.

Do ponto de vista econômico, uma das principais barreiras é a insuficiência de financiamento nacional que se associa à rigidez de limites fiscais, os quais dificultam a criação de mecanismos permanentes de investimento em infraestrutura e vigilância resiliente no SUS ^24^. Assim, é urgente a criação de um fundo nacional de adaptação, com alocações específicas para os setores de saúde, desenvolvimento urbano e proteção ambiental. 

Especificamente para o setor saúde, um Programa Nacional de Adaptação desse setor deve ser desenvolvido e implementado, cuja função seja mobilizar e distribuir recursos entre os níveis federal, estadual e municipal. Esses fundos devem incentivar as autoridades subnacionais a desenharem estratégias de adaptação territorializadas, que respondam às vulnerabilidades e capacidades específicas de cada localidade. As secretarias estaduais e municipais de saúde devem assumir a liderança na implementação descentralizada, avançando independentemente do ritmo do governo federal.

Caminhos para a adaptação

Metas claras de adaptação devem ser integradas ao planejamento de médio e longo prazos no Plano Nacional de Saúde - e devem ser preferencialmente alinhadas com o estabelecido no Plano Plurianual. Outro ponto é a incorporação de dados climáticos nos protocolos rotineiros de vigilância em saúde e de preparação dos serviços ^12^. Uma adaptação eficaz requer a integração entre a vigilância em saúde, a atenção primária, a atenção especializada, os sistemas educacionais, as instituições acadêmicas e a sociedade civil, de modo a permitir uma resposta coordenada e intersetorial que reflita a diversidade territorial do Brasil ^10^.

Algumas ações que fortalecem o processo de adaptação no SUS foram iniciadas. Em 2024, o Ministério da Saúde do Brasil instituiu um grupo de trabalho para a elaboração do Plano Setorial de Adaptação às Mudanças Climáticas. Em 2025, a organização continua desenvolvendo o Plano de Adaptação do Setor Saúde, que tem seus eixos centrais voltados à vigilância em saúde, atenção à saúde, promoção da saúde e ciência, tecnologia, inovação e produção do setor. 

Experiências de adaptação, que exercem impactos na saúde, não são exclusivas do setor de saúde e podem demandar negociações e políticas intersetoriais, envolvendo setores como o Ministério do Meio Ambiente e Mudança do Clima e o Ministério das Cidades, o que aumenta ainda mais a complexidade do processo. Intervenções voltadas para infraestrutura urbana, cidades verdes e sustentáveis e melhorias de saneamento e educação ambiental são ações já relatadas na literatura científica como contribuintes com a saúde da população em contextos de impactos por condições climáticas ^25^.

É imprescindível que se estabeleça uma agenda sólida, robusta e sustentável de vigilância em saúde informada pelo clima, com monitoramento contínuo de indicadores epidemiológicos e ambientais como temperatura, precipitação e níveis dos rios. As equipes locais de vigilância epidemiológica devem estar capacitadas para interpretar as relações entre variáveis climáticas e agravos e doenças, assim como antecipar eventos extremos como enchentes e secas de rios para um planejamento robusto e resposta oportuna.

Construir resiliência climática no SUS é um desafio de saúde pública urgente e complexo que o Brasil enfrenta e que perdurará por anos. Esse processo exige integração de dados meteorológicos, ambientais e epidemiológicos; planejamento multiescalar e de longo prazo; e investimentos estratégicos consistentes e contínuos.

Embora apoio político e liderança federal sejam essenciais para a mobilização de recursos e definição de diretrizes políticas, o núcleo de adaptação efetiva está no empoderamento de estados, municípios e sociedade civil como atores centrais. Sua capacidade de oferecer respostas rápidas e adequadas às populações socialmente vulneráveis é crucial.

Alcançar a resiliência climática exigirá compromisso político, planejamento territorial e colaboração intersetorial contínua para garantir o cumprimento do mandato constitucional do SUS e assegurar o direito à saúde diante de um clima em transformação.
